# Adamantane Containing Peptidoglycan Fragments Enhance RANTES and IL-6 Production in Lipopolysaccharide-Induced Macrophages

**DOI:** 10.3390/molecules25163707

**Published:** 2020-08-14

**Authors:** Mateja Manček-Keber, Rosana Ribić, Fernando Chain, Davy Sinnaeve, José C. Martins, Roman Jerala, Srđanka Tomić, Krisztina Fehér

**Affiliations:** 1 Department of Synthetic Biology and Immunology, National Institute of Chemistry, Hajdrihova 19, P.O. Box 660, SI-1001 Ljubljana, Slovenia; mateja.mancek@ki.si (M.M.-K.); roman.jerala@ki.si (R.J.); 2 Centre of Excelence EN-FIST, SI-1000 Ljubljana, Slovenia; 3 University Center Varaždin, University North, Jurja Križanića 31b, HR-42 000 Varaždin, Croatia; rosana.ribic@unin.hr; 4 Department of Organic and Macromolecular Chemistry, Ghent University, Krijgslaan 281 S4, 9000 Ghent, Belgium; fernando.chain@ugent.be (F.C.); Davy.Sinnaeve@ugent.be (D.S.); jose.martins@ugent.be (J.C.M.); 5 Univ. Lille, Inserm, Institut Pasteur de Lille, CHU Lille, U1167—Labex DISTALZ—RID-AGE—Risk Factors and Molecular Determinants of Aging-Related Diseases, F-59000 Lille, France; 6 CNRS, ERL9002—Integrative Structural Biology, F-59000 Lille, France; 7 Department of Chemistry, Faculty of Science, University of Zagreb, Horvatovac 102A, HR-10 000 Zagreb, Croatia; stomic@chem.pmf.hr; 8 Heidelberg Institute for Theoretical Studies, Schloss-Wolfsbrunnenweg 35, 69118 Heidelberg, Germany; 9 Molecular Recognition and Interaction Research Group, Hungarian Academy of Sciences, University of Debrecen, Egyetem tér 1, H-4032 Debrecen, Hungary

**Keywords:** peptidoglycan, immunostimulation, lipid incapsulation, adamantane, mannose

## Abstract

We report the enhancement of the lipopolysaccharide-induced immune response by adamantane containing peptidoglycan fragments in vitro. The immune stimulation was detected by Il-6 (interleukine 6) and RANTES (regulated on activation, normal T cell expressed and secreted) chemokine expression using cell assays on immortalized mouse bone-marrow derived macrophages. The most active compound was a α-D-mannosyl derivative of an adamantylated tripeptide with L-chirality at the adamantyl group attachment, whereby the mannose moiety assumed to target mannose receptors expressed on macrophage cell surfaces. The immune co-stimulatory effect was also influenced by the configuration of the adamantyl center, revealing the importance of specific molecular recognition event taking place with its receptor. The immunostimulating activities of these compounds were further enhanced upon their incorporation into lipid bilayers, which is likely related to the presence of the adamantyl group that helps anchor the peptidoglycan fragment into lipid nanoparticles. We concluded that the proposed adamantane containing peptidoglycan fragments act as co-stimulatory agents and are also suitable for the preparation of lipid nanoparticle-based delivery of peptidoglycan fragments.

## 1. Introduction

The innate immune system is based on the recognition of danger signals, such as the microbial components of pathogens. This is enabled by families of pattern recognition receptors (PRRs) expressed by macrophages and other immune cells [1]. PRRs recognize peptidoglycan (PGN), which is a major component of the bacterial cell wall and acts as a pathogen associated molecular pattern (PAMP). PRRs are classified into Toll-like receptors (TLRs), RIG-I-like receptors (RLRs), NOD-like receptors (NLRs), and C-type lectin receptors (CLRs) [2].

Muramyl dipeptide (MDP), *N*-acetylmuramyl-l-alanyl-d-isoglutamine, is the minimal essential structural unit of PGN responsible for its immunological activity by the stimulation of NOD2 receptors [3]. Structure–activity studies of the MDP derivatives and analogues suggest that l-Ala-d-isoGln pharmacophore is essential for the immunostimulatory properties. The introduction of lipophilic substituents into MDP analogues have been shown to increase its adjuvant activity [4,5,6] and enable the incorporation of compounds into lipid nanoparticles [5]. In this study, lipophilic MDP derivatives were used for the preparation of liposome vesicles made from egg yolk phosphatidylcholine and 1-palmitoyl-2-oleoyl-phosphatidylglycerol, and their size distribution (95–120 nm) and structures were analyzed by dynamic light scattering (DLS). Lipid molecules, due to their amphipathic character and physical−chemical properties, act as surface-active compounds and form micelles and liposomes of different sizes in aqueous solutions. Lipid nanoparticles as drug delivery systems are well-established [7] and have found widespread use in vaccinations given their ability to stimulate the immune system and induce both cellular and humoral immune responses [8]. Muramyl tripeptide phosphatidylethanolamine (MTP-PE), a lipophilic MDP derivative, promotes the immune response and is being clinically used in the form of liposomes for the treatment of osteosarcoma [9].

**Figure 1:**
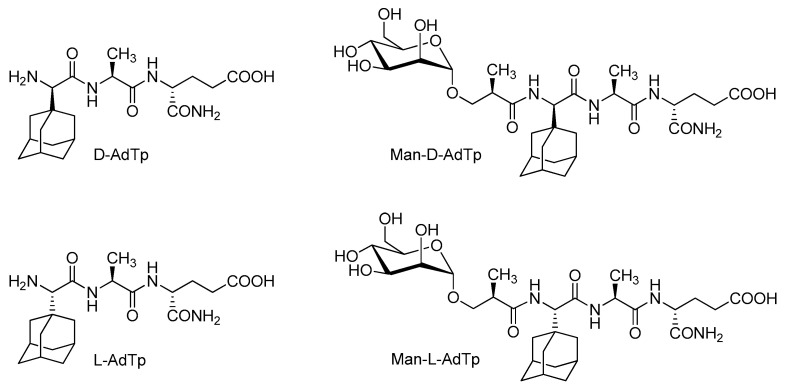
Chemical structures of investigated peptidoglycan (PGN) derivatives. d-AdTp: d-(adamant-1-yl)- Gly-l-Ala-d-isoGln, l-AdTp: l-(adamant-1-yl)-Gly-l-Ala-d-isoGln, Man-d-AdTp: (2*R*)-*N*-[3-(α-d- mannopyranosyloxy)-2-methylpropanoyl]-d-(adamant-1-yl)-Gly-l-Ala-d-isoGln, Man-l-AdTp: (2*R*)-*N*-[3-(α-d-mannopyranosyloxy)-2-methylpropanoyl]-l-(adamant-1-yl)-Gly-l-Ala-d-isoGln. The “Tp” notation stands for tripeptide.

Here, we focus on PGN derivatives shown in Figure 1 which have a minimal PGN fragment, l-Ala-d-isoGln, as cargo and a hydrophobic adamantyl group. Two adamantylated tripeptide (AdTp) diastereoisomers were investigated, d-AdTp and l-AdTp, that differ in stereochemistry of the adamantyl glycine attached at the N-terminus. The adamantyl group was introduced to increase the lipophilicity of the parent l-Ala-d-isoGln and to facilitate the anchoring of the PGN fragment cargo to the lipid bilayer as it is known that adamantane moiety interacts with lipid bilayer [10,11]. Both tripeptides were shown to be stable, non-pyrogenic, water soluble, non-toxic, and displayed an adjuvant effect in vivo in mice models [12].

Furthermore, conjugates of the tripeptides with mannose were also considered (Figure 1). The choice of the mannose was inspired by mannosylated liposomes, which have been shown to enhance the uptake and activation of dendritic cells and to increase T cell proliferation [13]. In this fashion, antigen presenting cells with expressing CLRs can be targeted by the mannosylated PGN derivatives. The mannosylation of the adamantyl compounds was indeed shown to further amplify the adjuvant activity in vivo, which might be related to the improved targeting of immune cells [11,12].

The composition of lipid nanocarriers, encapsulation efficiency, and size are factors that are important for the preparation of appropriate carrier formulations. All these factors can influence the physico–chemical features of liposomes, such as the lipid bilayer fluidity, which can in turn influence immune response [14]. For the lipid nanoparticle carriers, we used liposomes prepared from L-alpha-phosphatidylcholine (PC).

The immune stimulation of PGN compounds was examined using cell assays on immortalized mouse bone-marrow derived macrophages (iBMDMs) stimulated by lipopolysaccharide (LPS), which is commonly used for the induction of inflammation and the production of pro-inflammatory cytokines including chemokines. LPS activates innate immune responses, acting as PAMP for the Toll-like receptor 4 [15].

The immune stimulation was detected by Il–6 cytokine and RANTES chemokine production; Il–6 is secreted by macrophages and T cells to stimulate immune response in fighting infections, particularly in response to PAMPs, which are specific microbial molecules able to stimulate an innate immune response directly [16]. RANTES is an inflammatory chemokine, which is chemotactic for T cells, eosinophils, and basophils, and plays an active role in recruiting leukocytes into inflammatory sites [17].

## 2. Results

**Figure 2:**
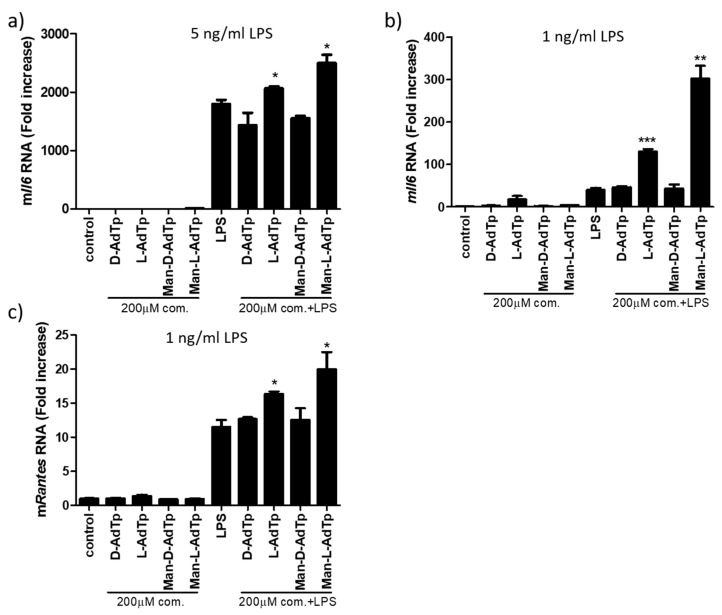
PGN derivates promote LPS signaling detected on the mRNA level. PGN derivatives (200 μM) were incubated or preincubated with iBMDMs and then stimulated with LPS for 4 h; for *Il6* with (**a**) 5 ng/mL LPS or (**b**) 1 ng/mL LPS and for *Rantes* (**c**) with 1 ng/mL LPS. RNA was isolated and qPCRs was performed. Statistics: Student’s *t*-Test * *p* < 0.05, ** *p* < 0.01, *** *p* < 0.001. As control unstimulated cells were used. Representative experiments from 3 independent experiments are shown.

The immunostimulatory properties of synthesized PGN compounds and the influence of lipid incorporation on the immunostimulation were investigated using in vitro cell assays on iBMDMs by the detection of Il–6 cytokine and RANTES chemokine. Their mRNA was quantified by qPCR (referred to as *Il6* and *Rantes*), while the corresponding protein levels (denoted as Il-6 and RANTES) were assessed using ELISA assay. We tested the potential activity of PGN compounds and compared the augmentation of immune stimulation by LPS with and without preincubation of the cells with the PGN derivatives. None of the compounds induced cytokine production (Figure 2a) nor pro-inflammatory NF-κB transcription factor (data not shown). When the cells were subsequently challenged with LPS, Man-l-AdTp strongly increased LPS induced *Il6* (* *p* < 0.05, Figure 2a) and *Rantes* (* *p* < 0.05, Figure 2c) mRNA production. In the case of the non-mannosylated derivatives, a significant (* *p* < 0.05) *Il6* and *Rantes* increase was detected for l-AdTp. Furthermore, when the concentration of LPS was lowered to 1 ng/mL, the contribution of the compounds to cytokine expression was higher (Figure 2b) further highlighting the contribution of Man-l-AdTp and l-AdTp.

**Figure 3:**
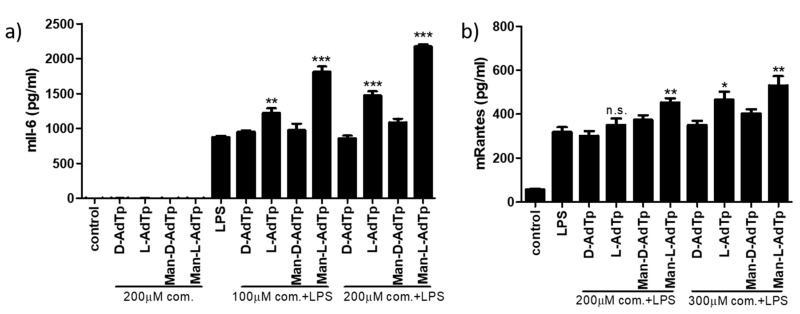
PGN derivates promote LPS signaling detected on the cytokine protein level. PGN derivatives (100 μM and 200 μM for Il-6 and 200 μM and 300 μM for RANTES) were incubated or preincubated with iBMDMs and then stimulated with LPS for 16 h; for Il-6 (**a**) with 5 ng/mL LPS and for RANTES (**b**) with 1 ng/mL LPS. ELISA assay was performed. Statistics: Student’s *t*-Test * *p* < 0.05, ** *p* < 0.01, *** *p* < 0.001. As control unstimulated cells were used. Representative experiments from 2 independent experiments are shown.

In order to confirm these qPCR results for mRNA, we performed ELISA assays to detect these cytokines on the protein level. We tested the compounds together with LPS at two concentrations for the detection of Il-6 and RANTES and alone for Il-6 (Figure 3). We observed similar patterns emerge for Il-6 in the ELISA assay as with the qPCR test at both concentrations of PGN compounds. There was no induction of Il-6 cytokines using the PGN compounds alone without LPS. When using the PGM compounds with LPS, Man-l-AdTp increased Il-6 levels to the largest extent (*** *p* < 0.001) and l-AdTp also induced significant increase (** *p* < 0.05 at 100 µM concentration and *** *p* < 0.001 at 200 µM concentration of applied PGM compound), while the D isomers did not affect the LPS induced Il-6 levels. The patterns observed for RANTES on the protein level confirmed the observation made with Il-6 ELISA assay showing increased stimulation by Man-l-AdTp (** *p* < 0.01 at both 200 and 300 µM concentration of applied PGM compound) and to lesser extent by l-AdTp (increase not significant at 200 µM concentration and * *p* < 0.5 at 300 µM concentration of applied PGM compound).

In order to investigate the influence of the lipid encapsulation of the adamantylated PGN derivatives and their mannose conjugates on their immune stimulation, phospholipid vesicles were prepared. The synthesized PGN derivatives were mixed with five-times excess of PC to prepare large phospholipid vesicles, which were then sonicated to break up the phospholipid suspension into small vesicles approximately 1 μm in diameter (Supplementary Figure S1) as determined by DLS.

**Figure 4:**
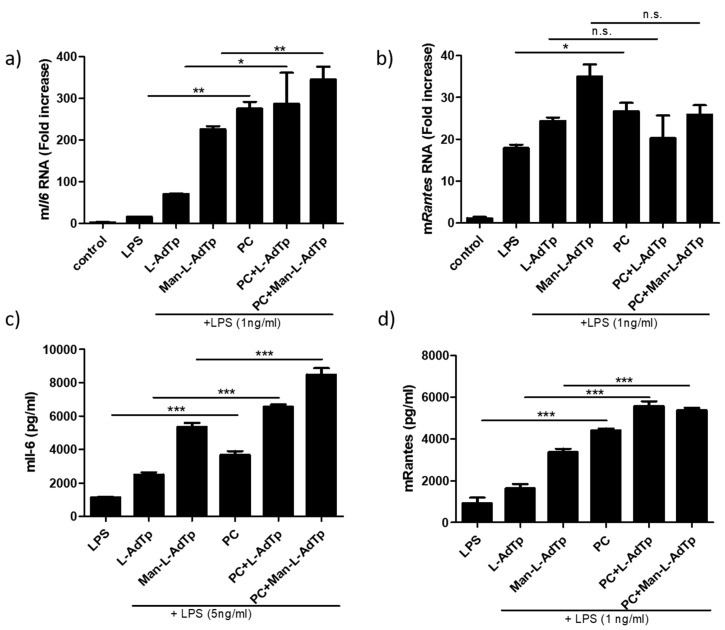
PGN derivatives incorporated into the lipid bilayer further augment immune responses as observed on the mRNA and protein level. (**a**) 1:5 PGN derivatives/PC mixtures were sonicated and DLS was measured. PGN derivatives (200 μM PGN derivatives/1mM PC mixtures) were preincubated for 1 h and then stimulated with LPS (1 ng/mL) for 4 h for qPCR and for 16h for the ELISA assay. RNA was isolated and qPCRs was performed; (**b**) for *Il6* and (**c**) for *Rantes*. ELISA assay was performed for Il-6 (**c**) with 5 ng/mL LPS and for RANTES (**d**). Statistics: Student’s *t*-Test * *p* < 0.05, ** *p* < 0.01, *** *p* < 0.001. Statistics: Student’s *t*-Test * *p* < 0.05, ** *p* < 0.01, *** *p* < 0.001, n.s.—not significant. As control unstimulated cells were used. Representative experiments from 3 independent experiments are shown.

The mixtures or PGN derivatives alone were preincubated with cells prior to LPS challenge. In this assay, we focused on the two most active compounds, l-AdTp and Man-l-AdTp. The mixture of PGN derivatives and PC promoted further cytokine expression after LPS stimulation as shown in Figure 4a,b. *Il6* enhancement was observed for l-AdTp (* *p* < 0.05) and for Man-l-AdTp (** *p* < 0.01), while the changes in *Rantes* were not conclusive.

We also tested the cytokine production of the lipid incorporated formulations on the protein level as shown in Figure 4c,d. Similarly to the qPCR results, an increase in Il-6 cytokine levels were observed with ELISA upon addition of both l-AdTp (*** *p* < 0.001) and Man-l-AdTp (*** *p* < 0.001) with Man-l-AdTp triggering a larger response. Detection of RANTES on the protein level clearly showed increased production of the chemokine upon addition of both l-AdTp (*** *p* < 0.001) and Man-l-AdTp (*** *p* < 0.001), both of which was not observable at the mRNA level.

## 3. Discussion

In this study we established an alternative methodology for testing the immune modulatory properties of peptidoglycan-based adjuvant candidates. While in vivo investigations in mice models [12,18,19] are invaluable in proving adjuvant properties, they are also expensive, time consuming, and require special facilities. The presented in vitro assays are simple, fast, cheaper, and can be performed in a simpler laboratory setting for the detection of the immune response induced by these compounds. These assays can be utilized in further studies on improved compounds or formulations.

The obtained results indicate that the tested PGN derivatives show immunostimulatory potential due to their l-Ala-d-isoGln core structure. The most active compound is the mannosylated derivative Man-l-AdTp, which strongly enhances production of LPS-induced proinflammatory cytokine and chemokine production with significant increase in Il-6 and RANTES both on the mRNA and the protein level. Non-mannosylated derivative l-AdTp also consistently, albeit to a lesser extent, enhanced Il-6 and RANTES production.

A similar stimulation of the immune response can be observed by MDP. MDP synergistically enhances the Toll-like receptor-induced production of cytokines, such as interleukin IL-1, tumor necrosis factor (TNF)-α, IL-6, and RANTES chemokine [20,21]. These results indicate that the chirality of adamantane containing amino acid influences the immunostimulatory properties and this effect is especially pronounced in mannosylated derivatives. In in vivo experiments performed in mice models using ovalbumin (OVA) as an antigen, described diastereoisomers of AdTp and their mannosylated derivatives exhibited adjuvant activities and differences regarding the configuration on stereogenic center of adamantyl glycine were also observed [12,18]. In mice of two genetically different strains, CBA (H-2k) and NIH/OlaHsd (H-2q), d-AdTp showed higher stimulation of anti-OVA IgG antibody production in comparison to l-AdTp [18]. The best adjuvant activity in NIH/OlaHsd (H-2q) mice showed the mannosylated derivate Man-d-AdTp [19]. Its good adjuvant activity was also confirmed in BALBc mice [22]. Differences noticed in vivo and in vitro regarding the stereocentre at the adamantyl group may arise because of the experimental model. In the in vivo experiments only the PGN derivatives were used to induce immune stimulation, while in the in vitro assay immune stimulation was induced by LPS, a TLR agonist, in combination with the PGN derivatives. The internalization processes and the complex intercellular communication, including PRR crosstalk, in the in vivo model may also result in differences in the immune responses. However, in both experimental models, the significant role of the mannose in the stimulation of immune response was confirmed. Mannose attachment can contribute to the targeting of mannose receptors. The mannose receptor (MR) on macrophages is a C-type lectin receptor (CLR), which facilitates the binding and internalization of microorganisms and glycoproteins with terminal mannose residues [23]. Recognition by the MR thus could increase uptake of the derivatives and thus contribute to their immune stimulation properties.

Previously, it has been shown that incorporation of a natural PGN fragment, a pentapeptide disaccharide, in egg-yolk phosphatidylcholine (PC) liposomes has increased its adjuvant activity as detected by secondary humoral immune response in vivo in mice [24]. We were interested in determining if the lipid encapsulation of the adamantylated PGN derivatives and their mannose conjugates, investigated here, could also promote their immune stimulation. The described in vitro experiments confirmed that the incorporation of the adamantane-containing PGN derivatives, l-AdTp and Man-l-AdTp, into lipid bilayers can enhance the immune stimulation. The lipid vehicles of PGN derivatives promoted cytokine expression after LPS stimulation. For l-AdTp and even more for Man-l-AdTp, a clear increase in Il-6 cytokine and RANTES was detected both on the mRNA and the protein level. The more pronounced enhancement of pro-inflammatory cytokine and chemokine expression by l-AdTp compared to Man-l-AdTp could be explained by the negative influence of the hydrophilic mannose group on the incorporation efficiency into phospholipid vesicles. NMR studies of the PGN derivatives in model lipid assemblies have indeed confirmed that the non-mannosylated compounds have larger lipid incorporation efficiencies and penetrate the bilayer deeper than compounds with mannose groups [25].

While the active lipophilic PGN derivatives with aromatic moieties and fatty acid chains incorporated into liposomes were extensively explored [5,6], adamantane group attachment to pharmacologically active fragments was also found to be a successful strategy to anchor derivatives to the bilayer and thus enhance immune responses [11]. A comprehension of adamantane distribution in the lipid bilayer is important to better understand the pharmacological profiles and mechanism of action of adamantane containing drugs, such as amantadine, rimantadine, and memantine [10]. Previous research on similar model PGN compounds with adamantyl-2-yl attachment incorporated into liposomes using atomic force microscopy revealed that the adamantane group is anchored into the lipid bilayer and that mannose residues are distributed at the surface of nanoparticles [26]. The localization and orientation of previously described adamantylated PGN derivatives in model lipid assemblies were also explored in detail using NMR spectroscopy, which showed the localization of bulky adamantane group in the interior of the bilayer and that of the D-iGln containing PGN fragment, as well as the presence of the mannose group on the surface of the bilayer [25].

Mannosylated liposomes preferentially target macrophages and dendritic cells, both in vitro and in vivo, and improve liposome internalization by macrophages and, consequently, enhance the immune response [25]. Hence, mannosylated nanoparticles formed by Man-l-AdTp could potentially be used for specifically targeting surface attached CLRs such as the MR. Receptor recognition of the mannosylated adamantylated PGN compounds, however, merits further investigation.

Combinations of PRR ligands, such as PGN fragments and LPS, enable NLR/TLR crosstalk resulting in the modulation of innate and adaptive immune responses [27]. The application of multi-PRR activation approaches can significantly increase immunity and presents a new approach for the design of novel vaccines [28]. Therefore, in the future, we envisage investigating interactions of non-mannosylated and mannosylated PGN derivatives with PRR receptors as well as driving forces governing the encapsulation of these derivatives into lipid-based delivery systems.

## 4. Materials and Methods

### 4.1. Chemicals and Materials

All reagents were purchased from Sigma-Aldrich. LPS (from Salmonella abortus equi HL83) was a gift from K. Brandenburg (Forschungszentrum Borstel, Germany). The tested compounds were prepared as previously described [11,19].

### 4.2. In Vitro Assay with QPCR

iBMDMs [29] from wt C57BL/6 were a gift from Prof. K. A. Fitzgerald (University of Massachusetts Medical School, Worcester, MA, USA). The cells were seeded (0.5 × 10^6^ cell/well) in DMEM + 5 % FBS (both Invitrogen) and stimulated or preincubated with PGN derivatives 1 h before LPS stimulation. l-alpha-phosphatidylcholine (l-alpha-PC) from egg yolk was dissolved in chloroform, evaporated, and dissolved in PBS. PGN derivatives were then mixed with five-time excess of l-alpha-PC and sonicated for 15 min (Elmasonic S 60H—Elma). PGN derivatives/PC mixtures were preincubated 1 h before LPS stimulation. After 4 h cells were lysed, and RNA was isolated according manufacturer’s instructions (Roche). cDNA was prepared using High capacity cDNA reverse transcription kit (Applied Biosystems) and qPCR for mouse *Gapdh* (Fw TTCACCACCATGGAGAAGGC; Rv GGCATGGACTGTGGTCATGA), *Il6* (Fw CGGAGGCTTAATTACACATGTTC; Rv CTGGCTTTGTCTTTCTTGTTATC), *Rantes* (Fw TCGTGCCCACGTCAAGGAGTATTT; Rv ACTAGAGCAAGCGATGACAGGGAA) was performed using SYBR green I master kit (Roche) on LightCycler 480 (Roche).

### 4.3. ELISA

iBMDMs were seeded at (1 × 10^5^ cells/well) in DMEM + 5 % FBS. The cells were preincubated with PGN derivatives or PGN derivatives/PC mixtures 1 h before LPS stimulation. After 16 h, Il-6 (Bioscience) and RANTES (Invitrogen) concentrations in supernatants were measured by ELISA according to manufacturer’s instructions. Absorbance was measured on SynergyMx (BioTek).

### 4.4. Dinamic Light Scattering

The size distribution of PGN derivatives/PC mixtures was measured on a ZetasizerNano (Malvern, UK) at 20 °C using an angle of 173° and 633-nm laser.

### 4.5. Statistical Analysis

GraphPad Prism 5 was used for preparing the graphs and performing statistical analysis. Representative experiments are shown. Data shown are the mean ± SEM. For the analysis of experimental data, *t*-test was used. When variance between repeats was high, a logarithmic (LN) transformation was applied for the data used in statistical tests.

## Supplementary Materials

The following are available online, Figure S1: Size distribution of the liposome preparation made from l-alpha-phosphatidylcholine (PC) after sonication.
