# Supplementary material for: Adamantane Containing Peptidoglycan Fragments Enhance RANTES and IL-6 Production in Lipopolysaccharide-Induced Macrophages
Source: Molecules. 2020 Aug 14;25(16):3707. doi: 10.3390/molecules25163707 (PMC7465286; doi:10.3390/molecules25163707)

Supplementary Information

Adamantane containing peptidoglycan fragments enhance RANTES and IL-6 production in lipopolysaccharide-induced macrophages

Mateja Manček-Keber [1,8,+], Rosana Ribić [2,+], Fernando Chain [3], Davy Sinnaeve [3,4,5], José C. Martins [3], Roman Jerala [1,8], Srđanka Tomić [6] and Krisztina Fehér [7,8*]

[1] Department of Synthetic Biology and Immunology, National Institute of Chemistry, Hajdrihova 19, POBox 660, SI-1001 Ljubljana, Slovenia; mateja.mancek@ki.si (M. M.-K.), roman.jerala@ki.si (R. J.)
[2] University center Varaždin, University North, Jurja Križanića 31b, HR-42 000, Varaždin, Croatia; rosana.ribic@unin.hr (R.R.);
[3] Department of Organic and Macromolecular Chemistry, Ghent University, Krijgslaan 281 S4, 9000, Ghent, Belgium; fernando.chain@ugent.be (F. C.), Davy.Sinnaeve@ugent.be (D.S.), jose.martins@ugent.be (J. C. M.)
[4] Univ. Lille, Inserm, Institut Pasteur de Lille, CHU Lille, U1167 - Labex DISTALZ - RID-AGE - Risk Factors and Molecular Determinants of Aging-Related Diseases, F-59000 Lille, France
[5] CNRS, ERL9002 - Integrative Structural Biology, F-59000 Lille, France
[6] Department of Chemistry, Faculty of Science, University of Zagreb, Horvatovac 102A, HR-10 000, Zagreb, Croatia; stomic@chem.pmf.hr
[6] Heidelberg Institute for Theoretical Studies, Schloss-Wolfsbrunnenweg 35, 69118 Heidelberg, Germany; feher.krisztina@h-its.org
[7] Molecular Recognition and Interaction Research Group, Hungarian Academy of Sciences, University of Debrecen, Egyetem tér 1, H-4032 Debrecen, Hungary
[8] Centre of Excelence EN-FIST, SI-1000 Ljubljana, Slovenia
* Correspondence: feher.krisztina@science.unideb.hu, feher.krisztina@h-its.org Tel.: +36 52 512900 Fax: +36 52 518 660

Figure S1 Size distribution of the liposome preparation made from L-alpha-phosphatidylcholine (PC) after sonication.

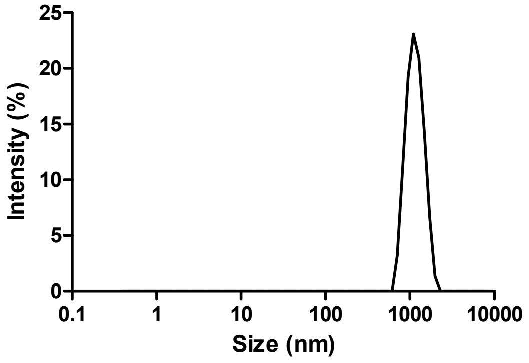

Supplement: Supplementary file 1 [file molecules-25-03707-s001.pdf]
